# Supplementary figures and images for: Mitochondrial genomes of Columbicola feather lice are highly fragmented, indicating repeated evolution of minicircle-type genomes in parasitic lice
Source: PeerJ. 2020 Mar 23;8:e8759. doi: 10.7717/peerj.8759 (PMC7098387; doi:10.7717/peerj.8759)

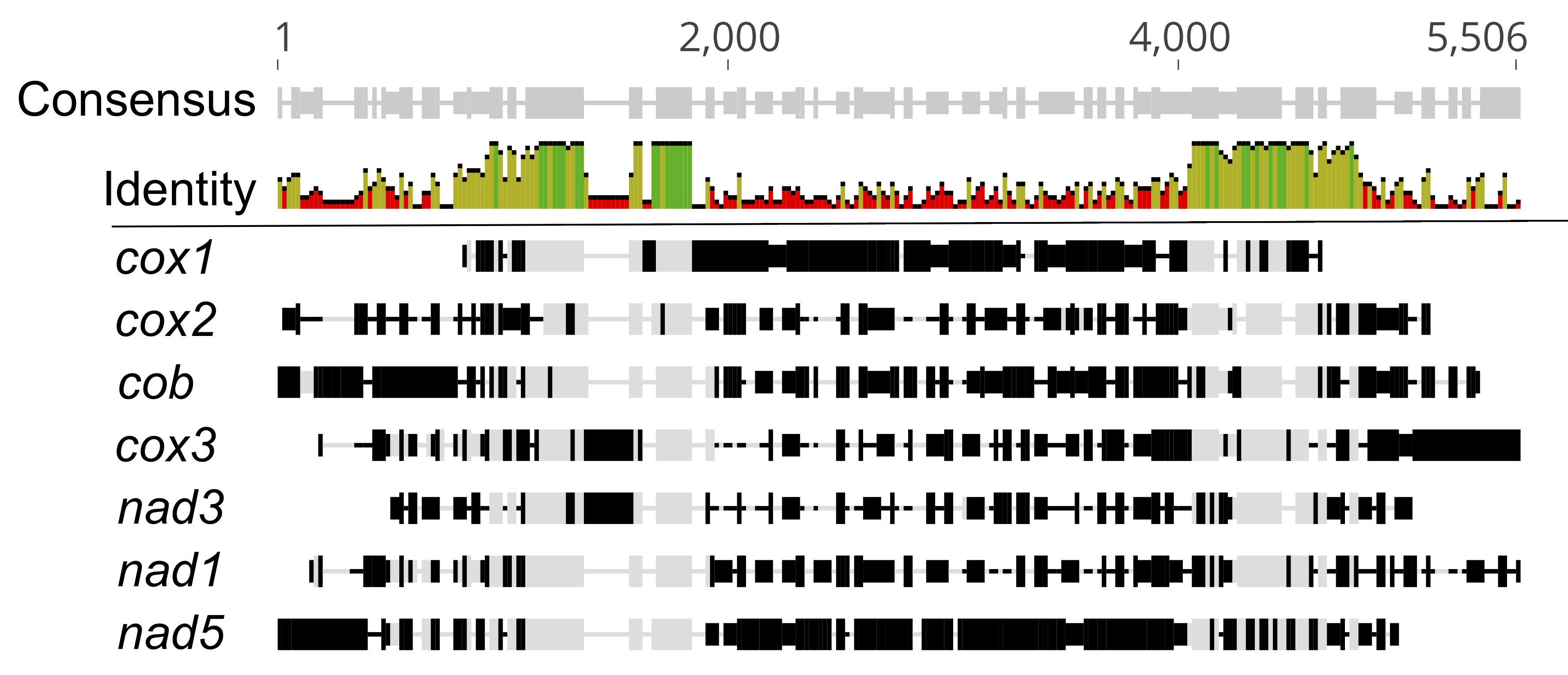

Supplement: Supplemental Information 1 — Contigs are labeled according to the protein-coding gene used as a reference. Identical sequences among the contigs are indicated with gray and variable sequences with black. [file peerj-08-8759-s001.jpg]

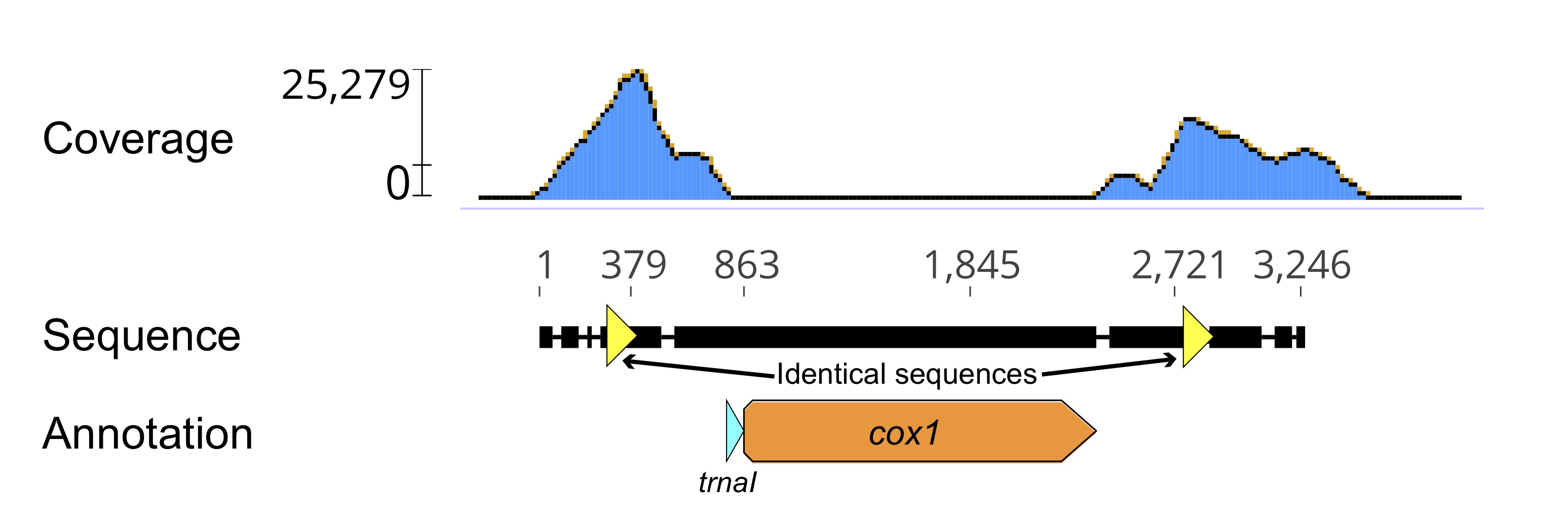

Supplement: Supplemental Information 2 — The contig was assembled in MITObim using cox1 from C. passerinae 2 (assembled from aTRAM) as a starting reference. Coding genes annotated from MITOS are indicated below the sequence. Regions of identical sequences are indicated with yellow triangles. Read coverage distribution was obtained by mapping reads used in MITObim to the assembled contig. [file peerj-08-8759-s002.jpg]

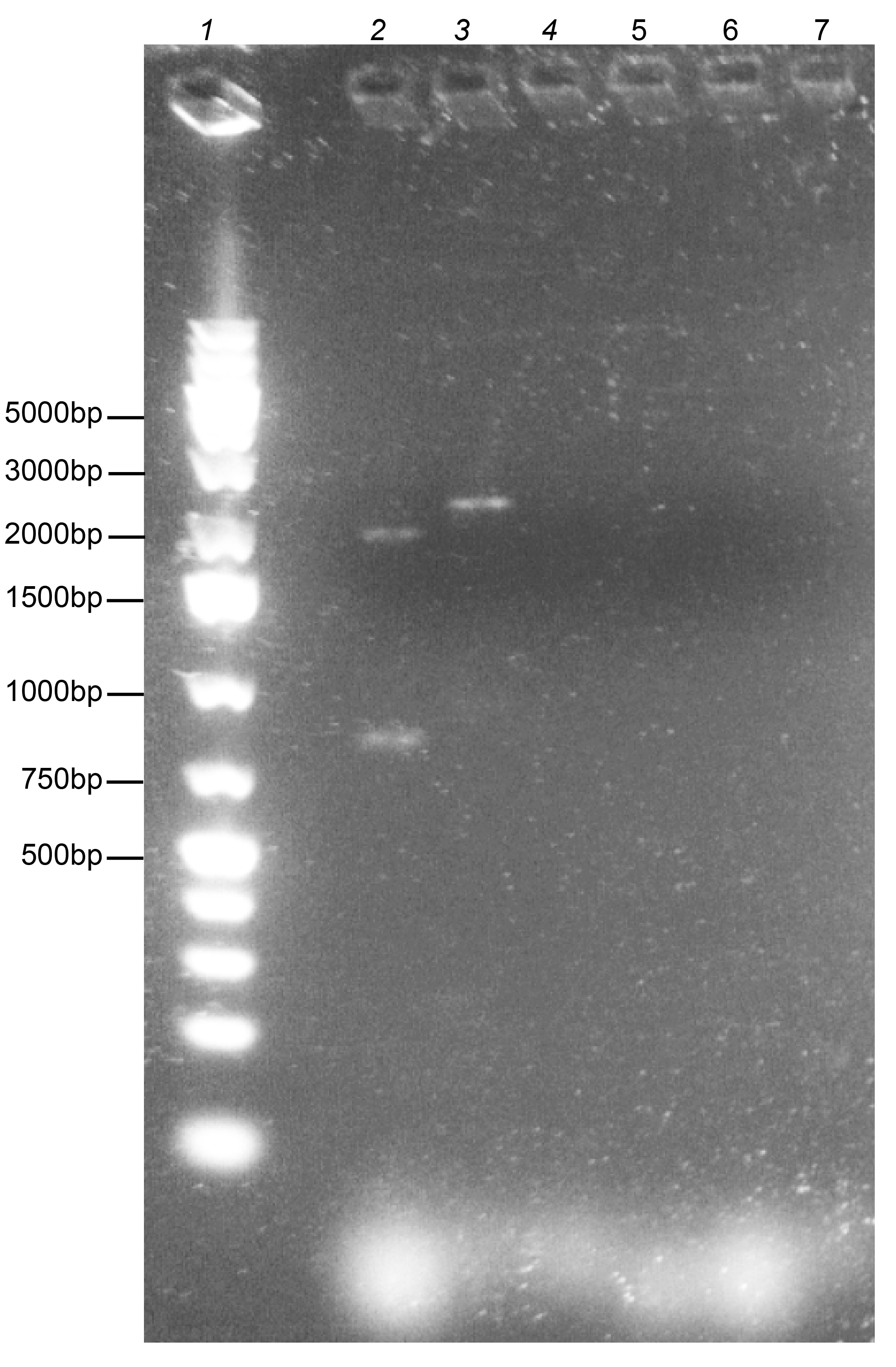

Supplement: Supplemental Information 3 — Lane 1: GeneRuler 1kb DNA Plus Ladder, Lane 2: primers CP2-12S-F and CP2-12S-R, Lane 3: primers CP2-COB-F and CP2-COB-R, Lane 4: primers CP2-12S-F and CP2-COB-R, Lane 5: primers CP2-12S-R and CP2-COB-F, Lane 6 and 7: negative controls for primer pairs CP2-12S-F/CP2-12S-R and CP2-COB-F/CP2-COB-F respectively. [file peerj-08-8759-s003.jpg]

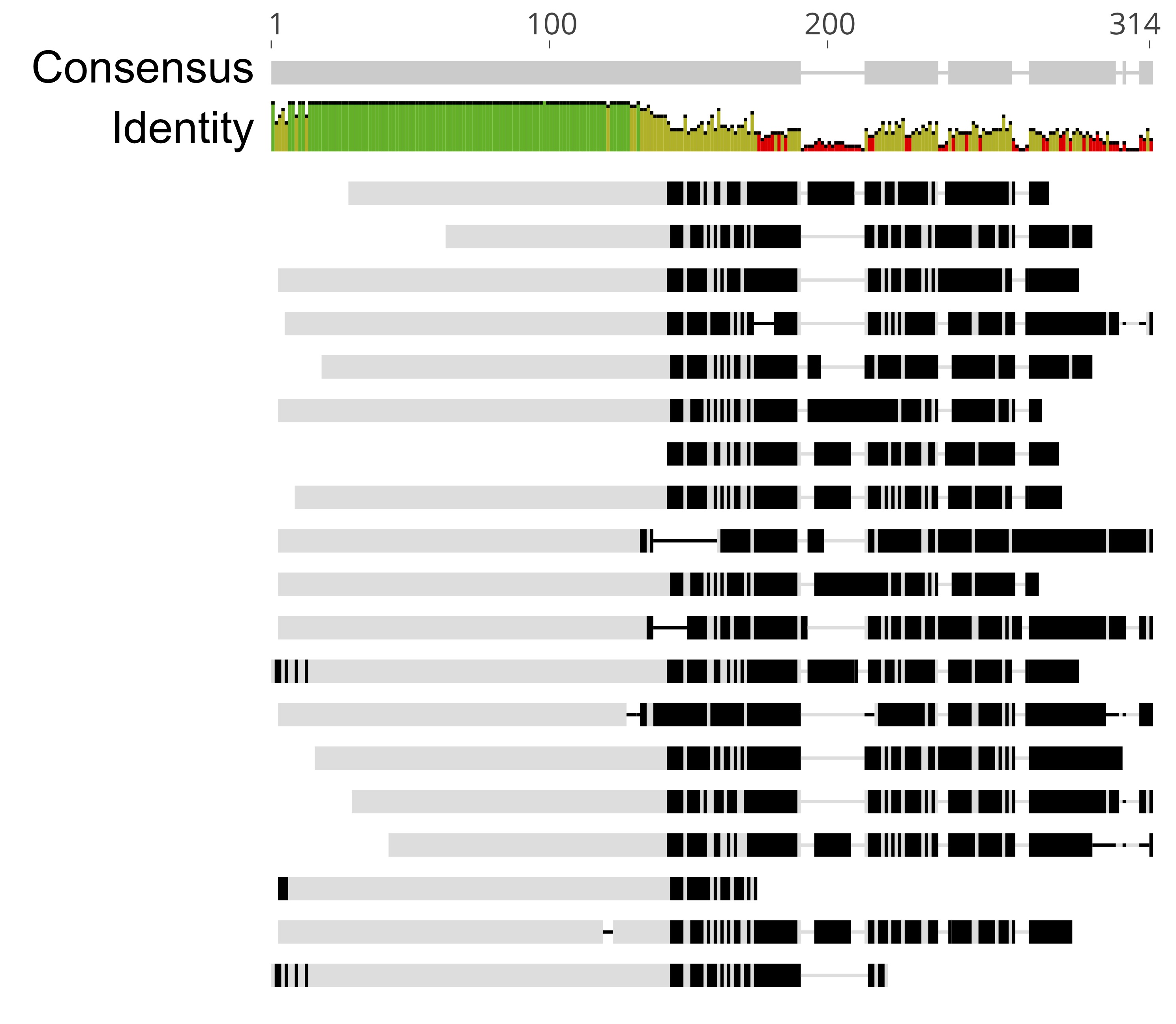

Supplement: Supplemental Information 4 — Identical sequences among contigs are indicated with gray and variable sequences with black. The variable regions were subsequently used as starting references in MITObim. [file peerj-08-8759-s004.jpg]

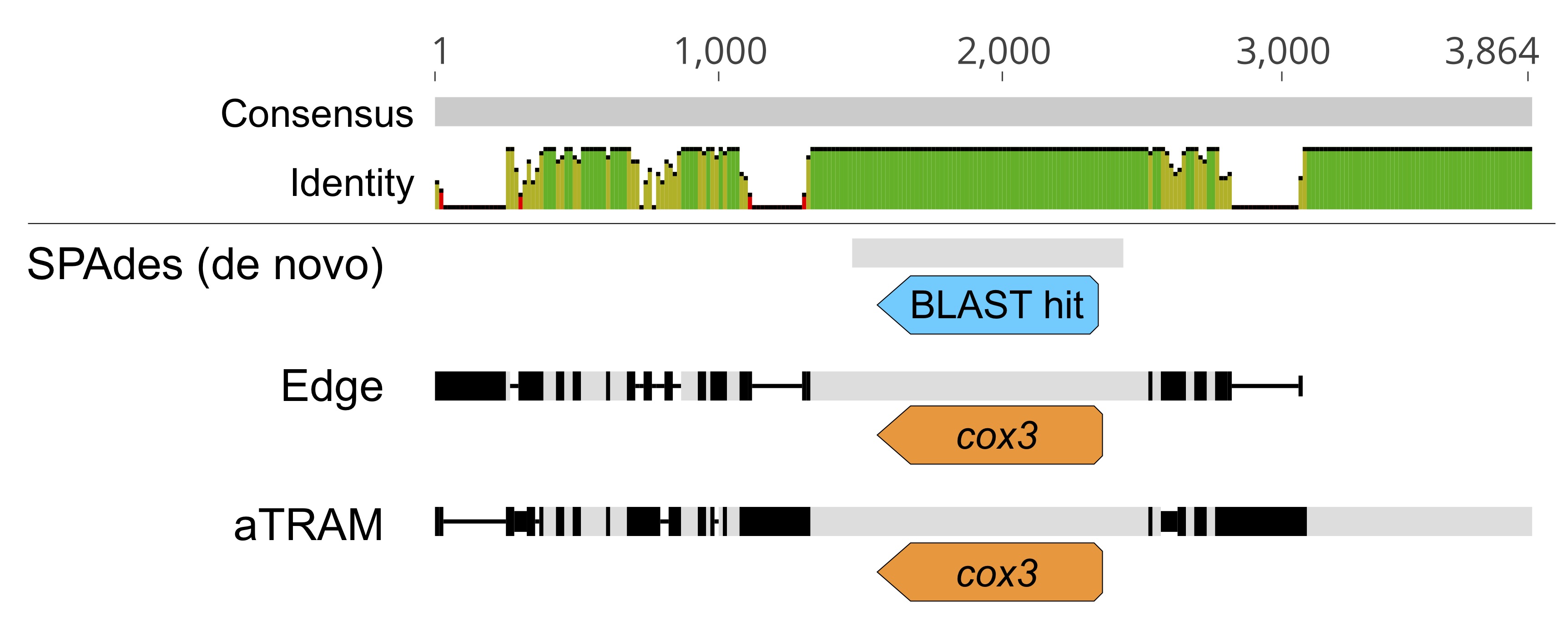

Supplement: Supplemental Information 5 — Gray indicates identical sequences among the contigs and black indicates variable sequences. Annotation from MITOS are shown below the Edge and aTRAM contigs (in this case cox3). The BLAST hit from searches with a cox3 query (Campanulotes compar) is shown below the de novo contig. [file peerj-08-8759-s005.jpg]

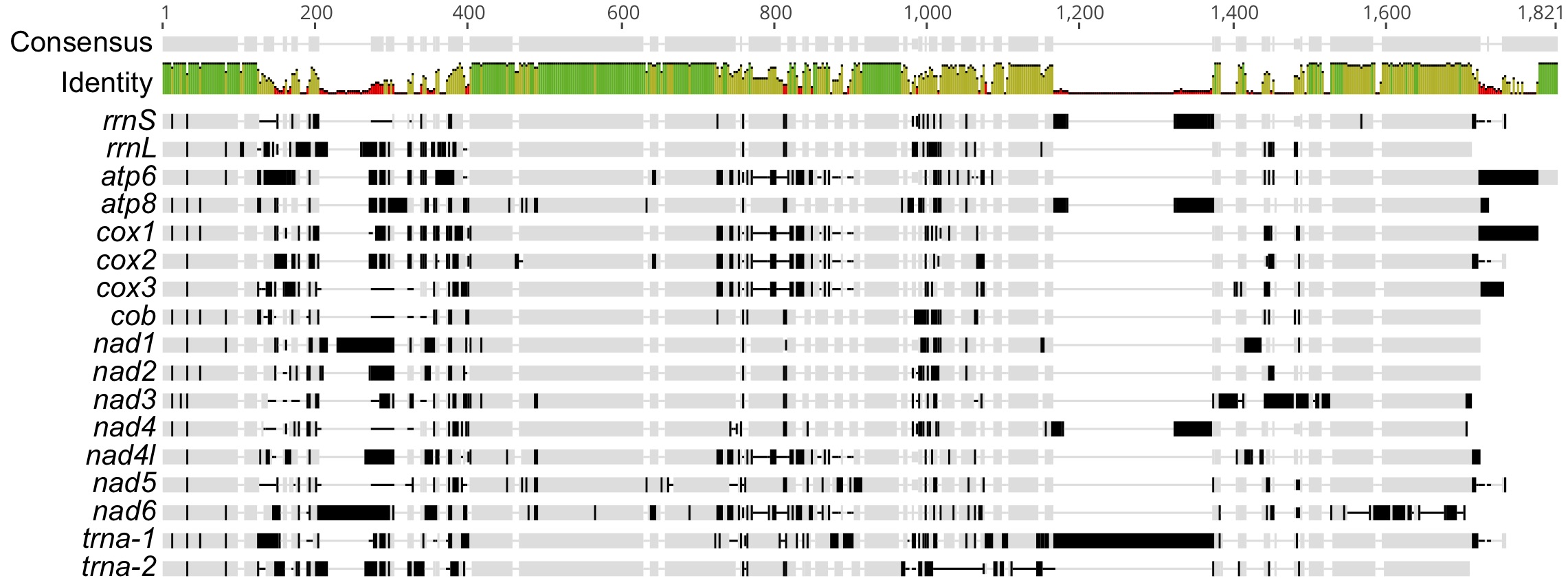

Supplement: Supplemental Information 6 — Names for each contig, labeled according to the gene content of each chromosome, are indicated to the left of the alignment. Gray indicates identical sequences among chromosomes and black indicates variable sequences. [file peerj-08-8759-s006.jpg]

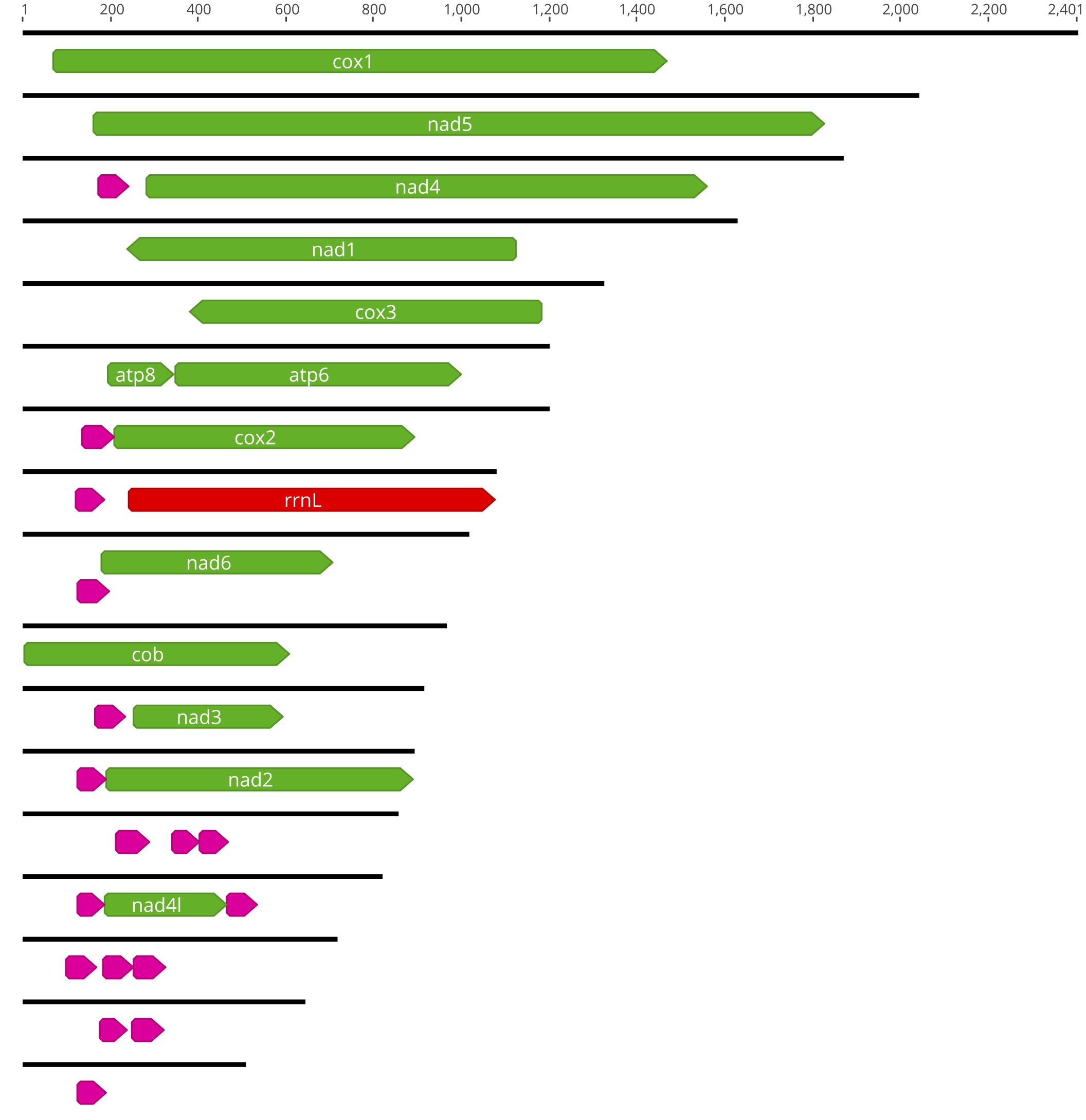

Supplement: Supplemental Information 7 — Each contig has been annotated with MITOS2 and trimmed according to published mitochondrial chromosomes of P. humanus. Protein-coding genes are indicated with green arrows, rRNA genes with red, and tRNA genes with pink. Lengths (bp) are indicated with the scale at the top of the figure. [file peerj-08-8759-s007.jpg]

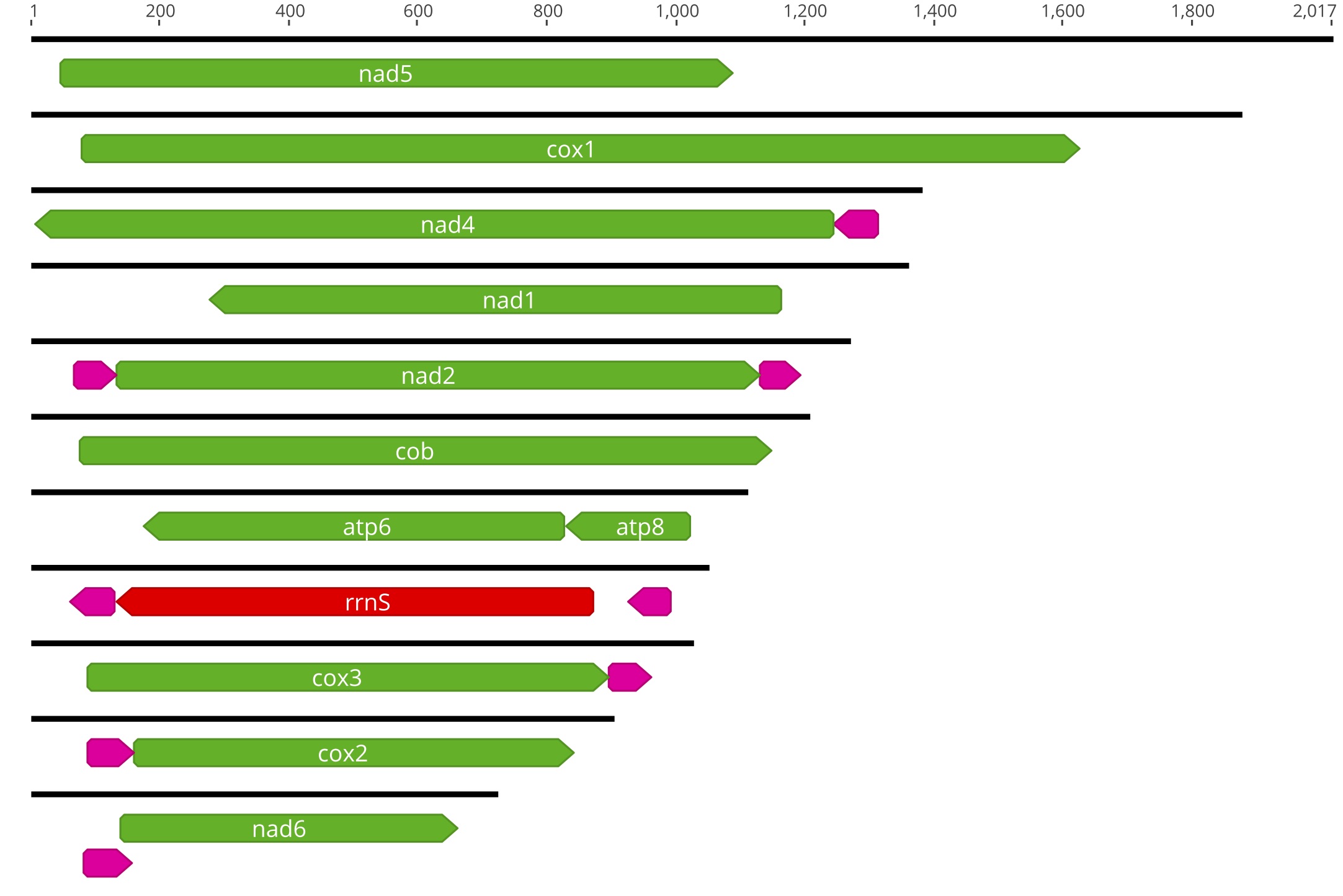

Supplement: Supplemental Information 8 — Each contig has been annotated with MITOS2. Protein-coding genes are indicated with green arrows, rRNA genes with red, and tRNA genes with pink. Lengths (bp) are indicated with the scale at the top of the figure. [file peerj-08-8759-s008.jpg]
