# Supplementary material for: Mitochondrial genomes of Columbicola feather lice are highly fragmented, indicating repeated evolution of minicircle-type genomes in parasitic lice
Source: PeerJ. 2020 Mar 23;8:e8759. doi: 10.7717/peerj.8759 (PMC7098387; doi:10.7717/peerj.8759)
Supplement: Article S1 [file peerj-08-8759-s014.pdf]

PCR confirmation of mitochondrial minicircles

We used polymerase chain reactions (PCR) to confirm the size and circularity of mitochondrial minicircles in *Columbicola passerinae* 2. First, we designed two pairs of outward-directed primers based on the assembled *rrnS* and *cob* genes (Table S2). Each 25µL PCR reaction contained 12.5 µL of Promega GoTaq Green Master Mix (Promega Corporation, Madison, WI, USA), 0.5 µL of each primer, 10.5 µL of deionized water, and 1 µL of genomic DNA. We set up reactions for each primer pair, including pairs of primers from different genes to confirm *rrnS* and *cob* are not on the same chromosome. We ran PCR with the following thermal cycler protocol: an initial denaturation step at 95°C for 2 minutes, 34 cycles of denaturation at 95°C for 30 seconds, annealing at 50°C for 30 seconds, and extension at 72°C for 3 minutes, followed by a final extension at 72°C for 5 minutes. We then ran the PCR products on a 1% agarose gel with GelGreen Nucleic Acid Gel Stain (Biotium, Fremont, CA, USA) and the GeneRule 1kb Plus DNA Ladder (Thermo Fisher Scientific, Waltham, MA, USA).
